# Supplementary material for: Quantum refrigeration powered by noise in a superconducting circuit
Source: Nat Commun. 2026 Jan 26;17:359. doi: 10.1038/s41467-025-67751-z (PMC12834946; doi:10.1038/s41467-025-67751-z)
Supplement: Supplementary file 1 — Supplementary Information [file 41467_2025_67751_MOESM1_ESM.pdf]

Supplemental material: Quantum refrigeration powered by noise in a superconducting circuit

I. EXPERIMENTAL SETUP

The experimental setup for our study is depicted in Fig. S1. Our device is placed at the mixing chamber stage of a dilution refrigerator, maintaining a stable temperature of 10 mK. To isolate from external interference's, the device is housed within a copper box which in turn is encased in a copper enclosure for electromagnetic wave shielding and a μ-metal enclosure for protection against low-frequency magnetic fields.

The routing of input and output signals is managed by a microwave circulator, enabling a reflection measurement setup to both the symmetric and antisymmetric waveguide. For the input lines going to our device, and to some extent the RF-line combining with the flux line going to qubit 1, we employ highly attenuated coaxial lines. The signals emanating from the device are then captured in output lines equipped with a High Electron Mobility Transistor (HEMT) amplifier at the 4 K stage. Additional amplifiers are used at room temperature (300K) to further amplify the signal.

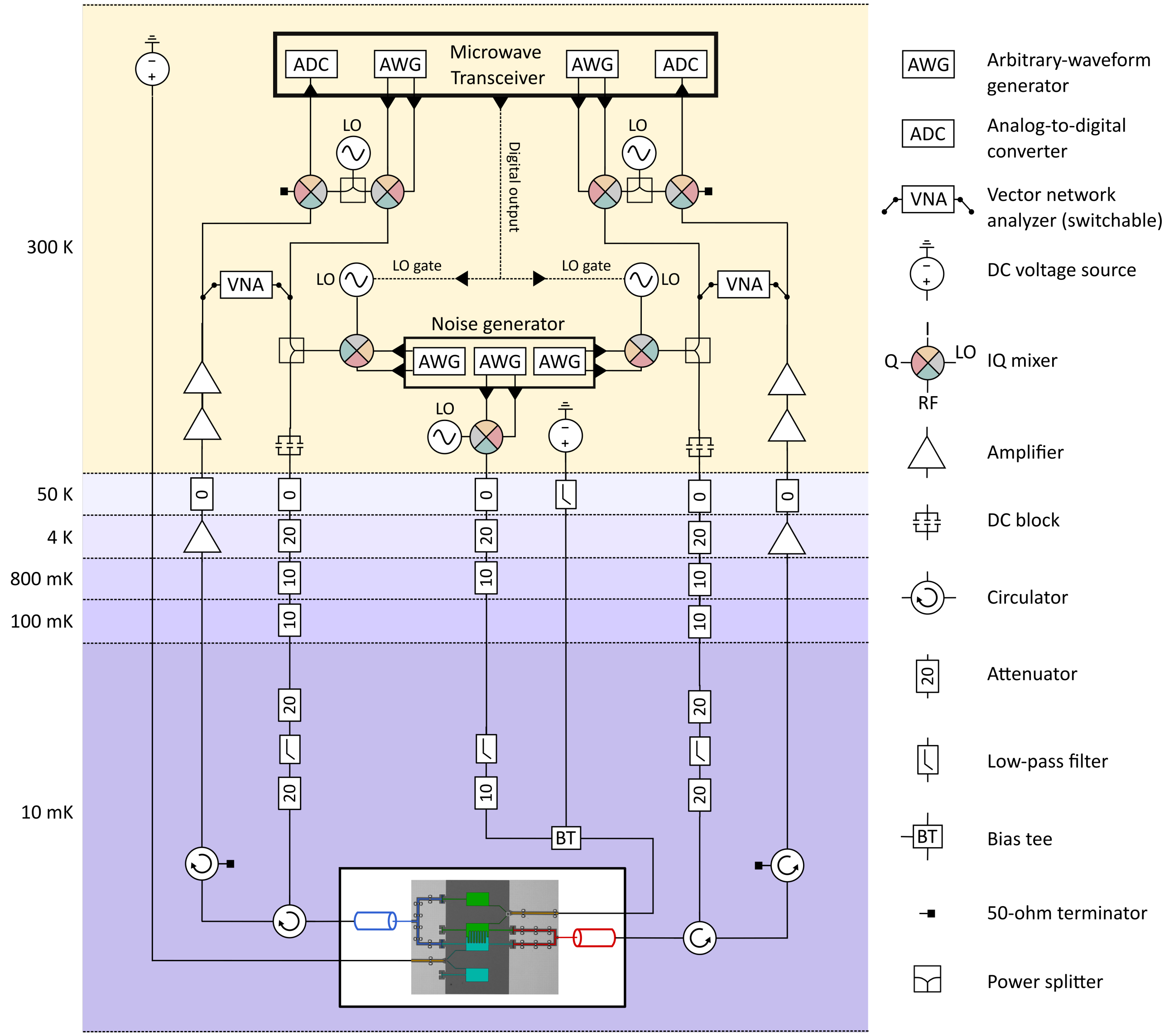

FIG. S1. Experimental setup, see text for description.

For our measurements we physically toggle between (indicated by a switch in Fig. S1) a vector network analyzer (VNA) and a microwave transceiver where the latter is used in conjunction with in-phase-quadrature (IQ) mixers. The VNA is used for continuous-wave reflection spectroscopy and the microwave transceiver for frequency-resolved measurements. For the microwave transceiver we utilize a Quantum Machines OPX+ which is comprised of both arbitrary waveform generators (AWG) and analog-to-digital converters (ADC). Their functionality are enhanced with FPGA logic, facilitating both interleaved measurements and math operations such as the ability to compute the power spectral density of a time signal.

To populate the waveguides with thermal noise we utilize arbitrary waveform generators of the Keysight 3202A model to continuously synthesize white voltage noise with a finite bandwidth of 30 MHz, a flat spectral density and centered at 200 MHz. The equipment is limited to a 500 MHz bandwidth so the noise is up-converted using IQ-mixers and local oscillators (Anapico APMS20G-4-ULN). The resulting continuous thermal radiation is segmented into pulses by gating the LO, coordinated by the digital outputs from the microwave transceiver playing simultaneously as the 5 μs readout events. This approach enables interleaved measurements of the power spectral density both with and without the applied noise. By doing so, the added noise can be directly subtracted from the background thermal radiation, thereby isolating the effects of our applied fields. When instead injecting coherent tones to the system, the AWG inside of the microwave transceiver are used. Similarly, the noise injected into the flux line going to qubit 1 is generated in the same way. It is combined with the DC current in the flux line using a cryogenic bias-tee.

II. RESPONSE TO DEPHASING NOISE

The reflection in the antisymmetric waveguide is examined under varying noise spectrum characteristics. White noise with a square spectral profile, a constant amplitude equivalent to $\Gamma_\phi/2\pi = 1.28$ MHz and a finite bandwidth of 50 MHz is generated. The center frequency ω_{CF} of the noise is varied around the energy difference $2g$ [Fig. S2(a)]. Notably, when the noise spectrum overlaps with the $2g$ frequency gap between the two modes, the linewidth of the $|0\rangle \to |a\rangle$ transition exhibits significant broadening. Furthermore, by increasing the noise bandwidth while maintaining a fixed amplitude (corresponding to $\Gamma_\phi/2\pi = 1.0$ MHz) and centering the frequency at $\omega_{CF} = 2g$ the linewidth saturates close after the bandwidth exceeds Γ_A [Fig. S2(b)].

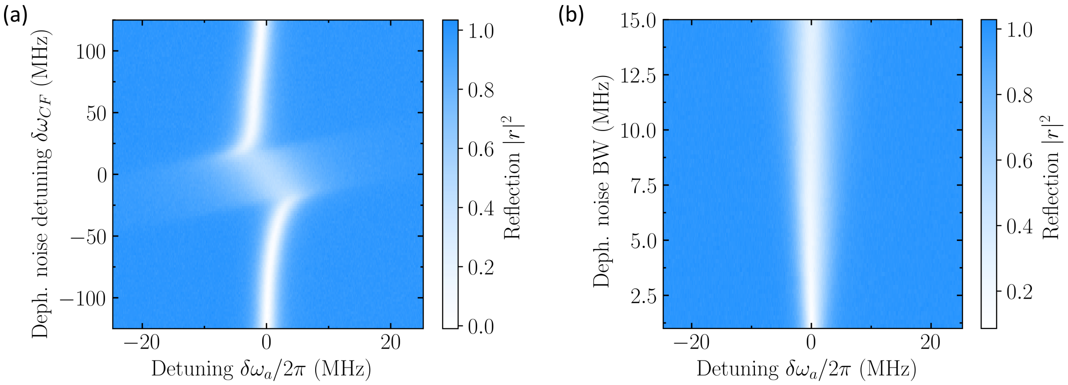

FIG. S2. Noise dependent reflection spectroscopy $|r|$ through the antisymmetric waveguide. (a) Reflection $|r|$ depending on the center frequency ω_{CF} of the applied noise for a fixed amplitude and a finite bandwidth of 50 MHz. (b) Reflection $|r|$ for increasing noise bandwidth for a fixed amplitude and $\omega_{CF} = 2g$.

III. THEORETICAL MODEL OF HEAT FLOWS WITH BATHS

In the case of two ideally hybridized qubits where $\omega_1 = \omega_2 = \omega$, the Hamiltonian of the system can be well approximated by

$$\mathcal{H} = \sum_{i=1,2} \omega\sigma_i^+\sigma_i^- + g\left(\sigma_1^+\sigma_2^- + \sigma_2^+\sigma_1^-\right). \tag{S1}$$

Here ω, σ_i^+ and σ_i^- are the bare mode frequency, creation and annihilation operators of qubit $i = 1, 2$ respectively and g is the inter-transmon coupling rate. When adding the anharmonicity α of the transmons as an additional term

TABLE S1. Eigenstates and eigenvalues of the diagonalized Hamiltonian. Analytical expressions found in reference [1].

Eigenstate	Bare states composition	Eigenvalue	Value/2 π
$\|0\rangle$	$\|0,0\rangle$	0	0GHz
$\|a\rangle$	$\|1,0\rangle - \|0,1\rangle$	$\omega - g$	5.305GHz
$\|s\rangle$	$\|1,0\rangle - \|0,1\rangle$	$\omega + g$	6.426GHz
$\|2+\rangle_L$	$\|2,0\rangle + \|0,2\rangle - \frac{\alpha+\sqrt{16g^2+\alpha^2}}{2\sqrt{2}g}\|1,1\rangle$	$\frac{1}{2}\left(4\omega + \alpha - \sqrt{16g^2+\alpha^2}\right)$	10.542GHz
$\|2-\rangle$	$\|2,0\rangle - \|0,2\rangle$	$2\omega + \alpha$	11.598GHz
$\|2+\rangle_U$	$\|2,0\rangle + \|0,2\rangle - \frac{\alpha-\sqrt{16g^2+\alpha^2}}{2\sqrt{2}g}\|1,1\rangle$	$\frac{1}{2}\left(4\omega + \alpha + \sqrt{16g^2+\alpha^2}\right)$	12.787GHz

to the Hamiltonian; $\sum_{i=1,2} \frac{\alpha}{2}\sigma_i^+\sigma_i^+\sigma_i^-\sigma_i^-$, the eigenstates and eigenvalues of the diagonalized Hamiltonian is found in the first- and second excitation manifold as presented in Table S1. From spectroscopy data we found $\omega/2\pi = 5.866$ GHz, $\alpha/2\pi = 133$ MHz and $g/2\pi = 560.1$ MHz when assuming identical transmons.

In the rotating frame, the creation and annihilation operators linked to the symmetric (S) and antisymmetric (A) modes are given by $\sigma_{\mathrm{s}}^{\pm} = \left(\sigma_1^{\pm} + \sigma_2^{\pm}\right)/\sqrt{2}$ and $\sigma_{\mathrm{a}}^{\pm} = \left(\sigma_1^{\pm} - \sigma_2^{\pm}\right)/\sqrt{2}$. The two waveguides couple to the $|0\rangle \to |s\rangle$ and $|0\rangle \to |a\rangle$ transitions with a rate Γ_s and Γ_a respectively, populated with a photon number denoted by n_s and n_a.

In the presence of dephasing of qubit 1, apart from the transverse coupling to the thermal reservoirs, the system exhibits longitudinal coupling to the spectral environment represented by $S_\phi(\omega)$ in the flux-line. This is done through the $\sigma_z^1 = \sigma_1^+\sigma_1^- - \sigma_1^-\sigma_1^+$ operator. Expressing the coupling in the symmetric- antisymmetric basis gives us

$$\sigma_z^1 = \frac{1}{2}(\sigma_z^s + \sigma_z^a) + \sigma_s^+\sigma_a^- + \sigma_a^+\sigma_s^-. \tag{S2}$$

Here the initial two terms represent pure dephasing where both the symmetric and antisymmetric modes "wobble" together with respect to the ground state. The rate at which this occur is proportional to the zero-frequency component $S_\phi(0)$ of the noise. In contrast, the following cross terms enable excitation exchange at a rate Γ_ϕ between the two modes, harnessing the frequency components at $S_\phi(2g)$ which bridges the energy gap between the two states. Because the noise injected into the flux line have a finite bandwidth of 50 MHz and centered at $2g$, the effect of the noise is well approximated by only taking the cross terms into account. The dynamics relevant for heat flow calculations between the reservoirs is described by the Lindblad master as described in the main text where the heat flows into the three separate channels is calculated to be

$$J_{j=\{s,a\}} \approx \frac{\hbar\left(n_a - n_s\right)\Gamma_a\Gamma_s\Gamma_\phi\left(g \pm \omega\right)}{\Gamma_s\Gamma_\phi + \Gamma_a\left(2\Gamma_s + \Gamma_\phi\right)} \tag{S3}$$

where the expression has been reduced to a linear expression of the populations through a first order expansion in n_s and n_a. Similarly;

$$J_\phi \approx -\frac{2g\hbar\left(n_a - n_s\right)\Gamma_a\Gamma_s\Gamma_\phi}{\Gamma_s\Gamma_\phi + \Gamma_a\left(2\Gamma_s + \Gamma_\phi\right)}. \tag{S4}$$

For the simulations shown in Fig. 4a in the main text, we use experimentally determined system parameters to obtain numerical values of the heat flows.

In the refrigeration region when the antisymmetric waveguide acts as the cold bath the coefficient of performance reads COP $= \frac{J_a}{J_s - J_a}$ compared to the Carnot limit to $\mathrm{COP}_{\mathrm{Carnot}} = \frac{T_a}{T_s - T_a}$ [2]. In the limit for large coupling rates and at the intersection between the heat engine and refrigeration regime, the COP for our device converges towards $\mathrm{COP}_{\mathrm{Carnot}}$ (Fig. S3).

IV. NUMERICAL SIMULATIONS OF DRIVEN SYSTEM

In the presence of a coherent drive with a frequency $\omega_d = \omega_s$ going into the symmetric waveguide, the governing Hamiltonian in the rotating frame can be expressed as:

$$\hat{\mathcal{H}} = \sum_{i=1,2}(\omega_i - \omega_d)\sigma_i^+\sigma_i^- + g\left(\sigma_1^+\sigma_2^- + \sigma_2^+\sigma_1^-\right) + \frac{\Omega_s}{2}\left(\sigma_s^+ + \sigma_s^-\right) \tag{S5}$$

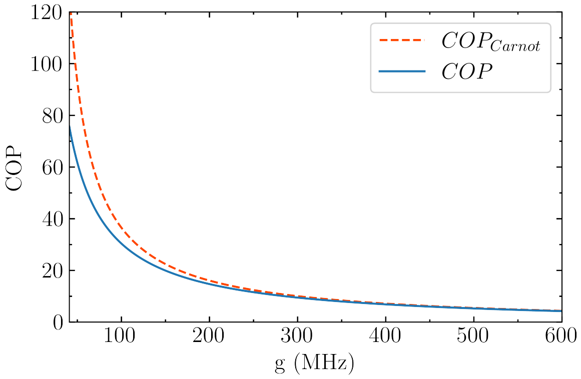

FIG. S3. Coefficient of performance (COP) for the device (blue) in the refrigeration regime and the Carnot limit $\mathrm{COP}_{\mathrm{Carnot}}$ as indicated by the dashed orange line.

The unitary dynamics are described by the Lindblad master equation in equation **??** but with the dissipators reflecting the absence of added thermal baths. Assuming negligible thermal population we have

$$\mathcal{L}_{j=\{s,a\}}\rho = \Gamma_j \mathcal{D}\left[\sigma_j^-\right]\rho. \tag{S6}$$

From the aforementioned definition of heat flow $J_a = \mathrm{Tr}\left(H\mathcal{L}_a\rho\right)$, the power transfer into the antisymmetric waveguide can be calculated as a function of dephasing. This is done using the python library QuTip 4.7.1 [3] through which the steady state density matrix can be calculated numerically. For the response in the symmetric waveguide, the power spectrum is calculated for the steady state solution when $\dot{\rho} = 0$ from a two-time correlation function

$$S(\omega) = \int_{-\infty}^{\infty} \langle A(\tau)B(0)\rangle e^{-i\omega\tau} d\tau. \tag{S7}$$

For the emission through the symmetric waveguide we use the correlation function $\langle\sigma_s^+(\tau)\sigma_s^-(0)\rangle$. By multiplying with the radiative decay rate Γ_s the correct magnitude is obtained, resulting in the power spectrum

$$S_s(\omega) = \Gamma_s \int_{-\infty}^{\infty} \langle\sigma_s^+(\tau)\sigma_s^-(0)\rangle e^{-i\omega\tau} d\tau. \tag{S8}$$

Using QuTip, this quantity is directly calculated and the emitted power is obtained by integrating $S_s(\omega)$ over a frequency span of 20 MHz. This is the same frequency span used in the measurements from the main text.

[1] M. A. Aamir, C. C. Moreno, S. Sundelin, J. Biznárová, M. Scigliuzzo, K. E. Patel, A. Osman, D. Lozano, I. Strandberg, and S. Gasparinetti, Engineering Symmetry-Selective Couplings of a Superconducting Artificial Molecule to Microwave Waveguides, Physical Review Letters **129**, 123604 (2022).

[2] L. Chen, Z. Ding, and F. Sun, A generalized model of an irreversible thermal Brownian refrigerator and its performance, Applied Mathematical Modelling **35**, 2945 (2011).

[3] J. R. Johansson, P. D. Nation, and F. Nori, QuTiP 2: A Python framework for the dynamics of open quantum systems, Computer Physics Communications **184**, 1234 (2013).
